# A Novel Case of Sudden Monocular Blindness Caused by Undiagnosed Granulomatous Disease Presenting with Acute Ophthalmic Artery Occlusion

**DOI:** 10.7759/cureus.6479

**Published:** 2019-12-27

**Authors:** Ryan C Gifford-Hollingsworth, Michael J Yoo, Zachary Sletten

**Affiliations:** 1 Emergency Medicine, San Antonio Military Medical Center, San Antonio, USA; 2 Emergency Medicine, Brooke Army Medical Center, Fort Sam Houston, USA

**Keywords:** ophthalmic, vision, emergency, granulomatous

## Abstract

Ophthalmic artery occlusion is a rare condition with a high morbidity, typically associated with cardiovascular disease and embolic or thrombotic phenomena. We present an atypical case of a 22-year-old active duty airman without comorbidities, who presented with acute, painless, monocular vision loss, found to have a right-sided, ophthalmic artery occlusion. The etiology for his vision loss was likely secondary to a granulomatous process at the orbital apex, causing compressive ischemia.

## Introduction

Ophthalmic and retinal artery occlusions are rare phenomena, occurring in approximately one to two in 100,000 patients [1,2]. Most commonly, these disease processes manifest as a sudden, painless vision or visual field defect in the ipsilateral eye [1,3]. In addition to these key components in the patient’s history, physical exam features that aid in diagnosis include decreased visual acuity, color vision changes or loss, and a relative afferent pupillary defect in the affected eye. Funduscopic exam may reveal retinal edema, box-carring, and retinal arteriolar attenuation. In contrast to retinal artery occlusions, ophthalmic artery occlusions typically lack a cherry red fovea but have profound optic disc edema [4]. Ultimately, an inpatient workup for both retinal and ophthalmic artery occlusions is indicated, as these conditions are considered to be stroke equivalents [5-7].

## Case presentation

A 22-year-old active duty airman with no past medical history presented to the emergency department (ED) for painless, unilateral, right-sided vision loss for one hour prior to arrival. During the patient’s morning physical fitness training, he noted an abrupt onset of small black spots and floaters in his right eye which progressed to globally decreased vision loss. Additionally, he noted only being able to see large movements at ranges less than one foot away. Further history revealed two similar episodes of floaters in his vision which was transient for minutes but also during physical exertion.

On arrival to the ED, the patient’s vitals included blood pressure of 138/69 mmHg, heart rate of 82 beats per minute, respiratory rate of 16 breaths per minute, pulse oximetry of 99% on room air, and an oral temperature of 99.0°F. The patient’s physical exam revealed intact extraocular movements, a visual acuity of 20/30 in his left eye, and visual acuity of shadows at close range in his right eye, with which he was only able to detect gross movements. Additionally, a right-sided significant relative afferent pupillary defect was noted in the patient’s affected eye. The patient otherwise had a non-focal neurologic exam. On review of systems, the patient endorsed right-sided vision loss but denied headache, pain with extraocular movements, numbness, tingling, fevers, chills, or head trauma. Workup in the ED, including intraocular pressure testing, non-dilated funduscopic exam, bedside retinal ultrasound, and a contrasted computed tomography of the head and neck, did not reveal a source for his monocular vision loss, which was characterized by retinal detachment, glaucoma, intracranial bleed or tumor, and cervical vascular pathology. Ophthalmology was consulted and performed a dilated eye exam which was concerning for optic neuritis versus a retinal artery occlusion.

**Figure 1:**
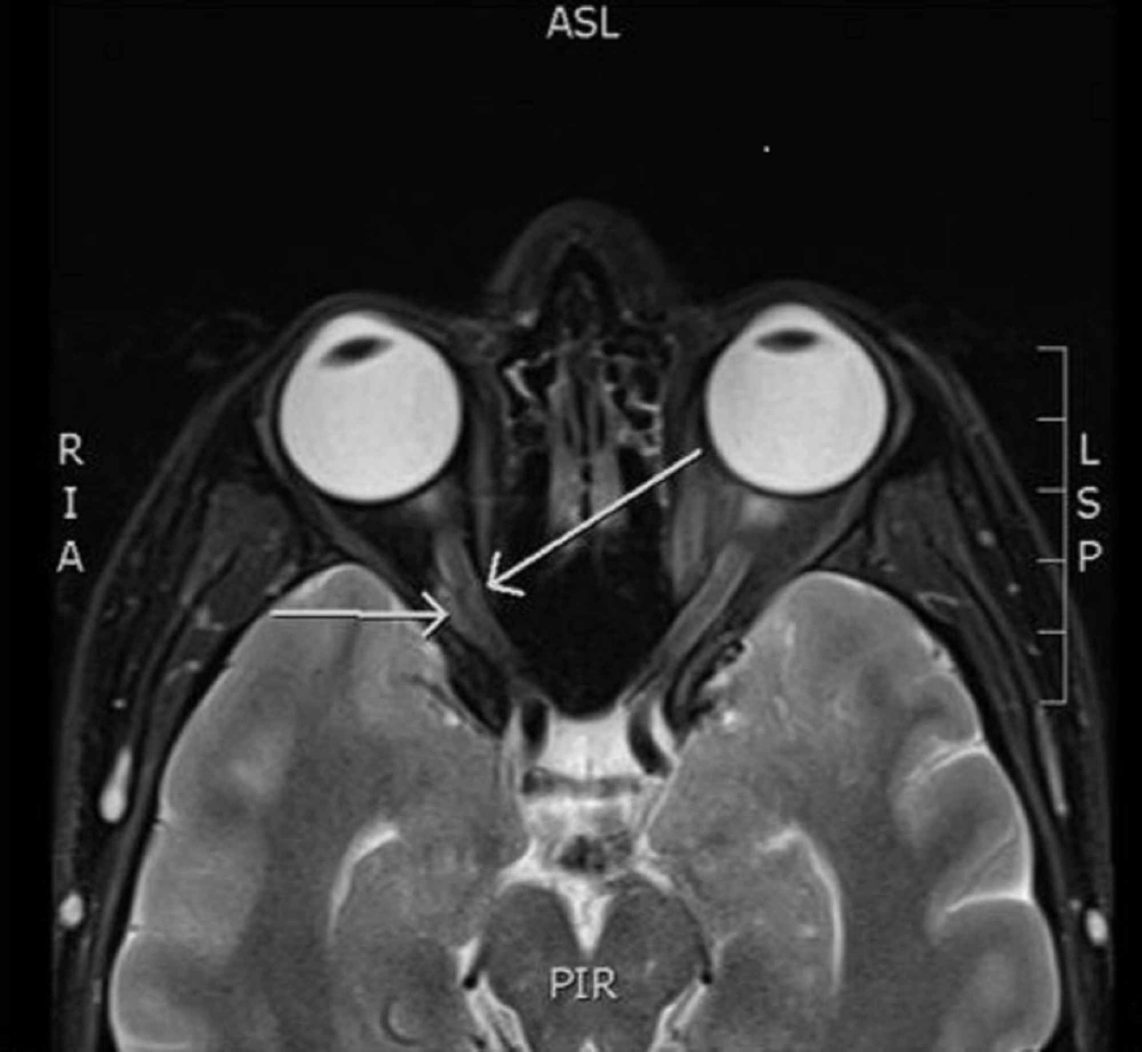
A transverse slice of a T2-weighted magnetic resonance image of the brain and orbits demonstrating right optic nerve sheath edema (white arrows).

**Figure 2:**
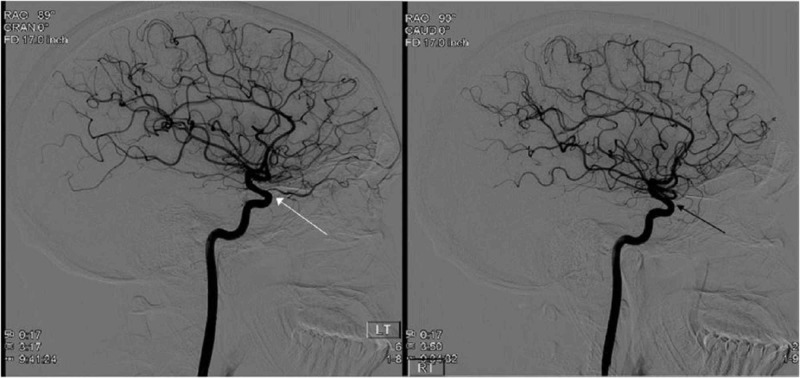
A bilateral carotid angiogram demonstrating the patient's normal left retinal artery (white arrow) in comparison to the right retinal artery occlusion (black arrow).

During admission, the patient had a repeat dilated eye exam which remained consistent with central retinal artery occlusion with choroidal hypoperfusion. An extensive coagulopathy workup, including a transthoracic echocardiogram and bilateral lower extremity ultrasounds, did not suggest an embolic etiology. A magnetic resonance imaging study of the brain and orbits demonstrated mild right optic nerve sheath edema secondary to a likely granulomatous process at the base of the optic nerve (Figure 1). The patient was started on 1 g of intravenous solumedrol daily, homatropine ophthalmic drops, and multiple hyperbaric treatments with minimal response. Further imaging with a bilateral carotid cerebral angiogram was concerning for compressive ischemia of the right ophthalmic artery (Figure 2). To further differentiate the source of his compressive findings, a computed tomography of the chest was performed, which demonstrated a tree-bud opacity in the left upper lung, further indicating that a systemic granulomatous disease process was the likely etiology. Unfortunately, the patient’s vision loss did not recover, and the workup continued in the outpatient setting.

## Discussion

Ophthalmic artery occlusion is a rare condition, typically associated with advanced age, systemic vascular disease, cardiovascular risk factors, inflammatory conditions, and embolic phenomena [1,3,5-7]. These associations remain consistent across all age groups, but patients under the age of 40 years have an increased incidence of congenitally acquired hypercoagulable states compared to the acquired forms seen in older populations [5,6]. Regardless of age, most cases of ophthalmic and retinal artery occlusions result in visual loss and retinal damage that is extensive and rarely reversible [1,3]. Although many methods of treating these occlusions have been trialed, few have shown consistent success [1]. These treatments include systemic medications (acetazolamide, mannitol, pentoxifylline, isosorbide dinitrate), topical glaucoma medications (latanoprost, timolol, acetazolamide), direct thrombolysis, systemic thrombolysis, ocular massage, hyperventilation, and hyperbaric therapy [1]. In most cases of ophthalmic and retinal artery occlusions, improving outcomes is directly related to early identification, long-term risk management, and medical optimization. The time at which the occlusion can be reversed with minimal to no permanent damage has been estimated from primate trials at 100 minutes, with massive and irreversible damage predicted at 240 minutes [3,8,9]. These data have been extrapolated to humans to recommend treatment and attempts at reperfusion within three hours, with consideration of acute treatment for up to six hours or longer depending on the clinical scenario [1].

Compression of the ophthalmic artery leading to acute monocular vision loss is a rare and minimally reported phenomenon. Current literature on compressive occlusion of the ophthalmic artery or its branches is sparse and limited to case reports or small case series. The cause is typically reported as prolonged prone positioning with direct external optic pressure or ophthalmic artery aneurysms [10,11]. Although granulomatous diseases, sarcoidosis and granulomatosis with polyangiitis, have a significant association with ophthalmic disease, retinal vascular occlusive disease is rare [12,13]. An extensive literature review revealed only one other reported case of a central retinal artery occlusion related to these granulomatous diseases [13,14]. To our knowledge, this is the first report of an ophthalmic artery occlusion related to a previously undiagnosed granulomatous disease.

In all previously reported cases of granulomatous retinal artery and vein occlusions, the authors theorized that the occlusion occurred secondary to the compressive nature of the granulomas [13,14]. Additionally, patterns of multiple preceding events of unilateral visual disturbances have been published [13,14]. We theorize that in the above case, exercise-induced vasospasm in the setting of an undiagnosed granulomatous disease exacerbated a known phenomenon of macular and optic nerve hypoperfusion [15].

In the ED, loss of eyesight is treated with the same urgency as the potential loss of limb or life. Vision loss, whether transient or permanent, requires emergency physicians to consider ophthalmic and retinal artery occlusions. Though this is especially true in elderly, comorbid patients, these conditions must continue to be in the differential diagnosis of even otherwise healthy patients presenting with acute monocular vision loss. Vision loss should be treated as equivalents to strokes or transient ischemic attacks, necessitating admission for an aggressive workup and medical optimization.

## Conclusions

Ophthalmic and retinal artery occlusions are rare conditions, associated with a high morbidity whose treatment outcomes are poor but improved by early recognition. These conditions should be suspected in any patient presenting with monocular vision loss, especially in those with a congenital or acquired hypercoagulable state. Prompt initiation of treatment is key in improving outcomes, as well as appropriately identifying and treating the underlying cause of the occlusive disease to prevent further morbidity and reducing the chances for permanent vision loss.
